# β2-microglobulin gene duplication in cetartiodactyla remains intact only in pigs and possibly confers selective advantage to the species

**DOI:** 10.1371/journal.pone.0182322

**Published:** 2017-08-16

**Authors:** Thong Minh Le, Quy Van Chanh Le, Dung Minh Truong, Hye-Jeong Lee, Min-Kyeung Choi, Hyesun Cho, Hak-Jae Chung, Jin-Hoi Kim, Jeong-Tae Do, Hyuk Song, Chankyu Park

**Affiliations:** 1 Department of Stem Cell and Regenerative Biology, Konkuk University, Hwayang-dong, Seoul, Republic of Korea; 2 Department of Molecular Science and Technology, Ajou University, Suwon, Republic of Korea; 3 Animal Biotechnology Division, National Institute of Animal Science, Rural Development Administration, Wanju-gun, Jeollabuk-do, Republic of Korea; University of Lausanne, SWITZERLAND

## Abstract

Several β2-microglobulin (B2M) -bound protein complexes undertake key roles in various immune system pathways, including the neonatal Fc receptor (FcRn), cluster of differentiation 1 (CD1) protein, non-classical major histocompatibility complex (MHC), and well-known MHC class I molecules. Therefore, the duplication of *B2M* may lead to an increase in the biological competence of organisms to the environment. Based on the pig genome assembly SSC10.2, a segmental duplication of ~45.5 kb, encoding the entire B2M protein, was identified in pig chromosome 1. Through experimental validation, we confirmed the functional duplication of the *B2M* gene with a completely identical coding sequence between two copies in pigs. Considering the importance of B2M in the immune system, we performed the phylogenetic analysis of *B2M* duplication in ten mammalian species, confirming the presence of *B2M* duplication in cetartioldactyls, like cattle, sheep, goats, pigs and whales, but non-cetartiodactyl species, like mice, cats, dogs, horses, and humans. The density of long interspersed nuclear element (LINE) at the edges of duplicated blocks (39 to 66%) was found to be 2 to 3-fold higher than the average (20.12%) of the pig genome, suggesting its role in the duplication event. The *B2M* mRNA expression level in pigs was 12.71 and 7.57 times (2^-ΔΔCt^ values) higher than humans and mice, respectively. However, we were unable to experimentally demonstrate the difference in the level of B2M protein because species specific anti-B2M antibodies are not available. We reported, for the first time, the functional duplication of the *B2M* gene in animals. The identification of partially remaining duplicated *B2M* sequences in the genomes of only cetartiodactyls indicates that the event was lineage specific. *B2M* duplication could be beneficial to the immune system of pigs by increasing the availability of MHC class I light chain protein, B2M, to complex with the proteins encoded by the relatively large number of MHC class I heavy chain genes in pigs. Further studies are necessary to address the biological meaning of increased expression of *B2M*.

## Introduction

Gene duplications provide resources for the random mutation, genetic drift, and selection, leading to an increase in the biological competence of organisms to the changing environment by increasing the buffering capacity of their genomes [1]. They also contribute to the phenotypic variations of species or populations [2,3]. The reported examples of gene duplication include genes with diverse biological roles, such as stomach lysozyme [4], visual pigment genes [5], homeobox family [6], hemoglobin gamma [7], alcohol dehydrogenase [8], ion channel family [9], and growth hormone family [10]. In mammals, the other classical examples include the duplication and dramatic expansion of the olfactory receptor (OR) genes to sense chemicals and *MHC* genes to recognize pathogens from diverse environments [11–13].

The copy number variations (CNVs) of *MHC* genes also affect the disease response status of individuals. For instance, the association of CNVs in *MHC* class I genes with Marek’s disease resistance was reported in chicken [14]. Additionally, a duplication of the *CIITA* gene, which encodes a trans-activator of the MHC class II molecules, was associated with the increased resistance to ingested nematodes in cattle [15]. Variations in the genomic structure of the human leukocyte antigen (HLA) due to the segmental duplications among haplotypes served as an important source of genetic diversity in relation to diseases [16].

β2-microglobulin, a protein with a molecular weight of 11.8 kDa, is ubiquitously expressed in all nucleated cells and present in most of the biological fluids, including serum, urine, and synovial fluid, in vertebrates [17,18]. The secondary structure of B2M possesses the characteristics of the immunoglobulins with a classical β-sandwich, which consists of two β-sheets linked by a single disulfide bridge [19,20]. B2M functions as a light chain in the heterodimer complexes with MHC class I and MHC class I-like heavy chains by noncovalent linkage [19,21]. Several B2M-bound protein complexes undertake key roles in various immune system pathways, including the neonatal Fc receptor (FcRn) [22], cluster of differentiation 1 (CD1) protein [23], human hemochromatosis protein (HFE) [24], mouse Qa (non-classical MHC) [25], and most well-known MHC class I molecules [26].

Recently, certain levels of the free B2M in blood have been shown to be associated with several cancers [27,28], age-related cognitive dysfunction, and impairment of neurogenesis [29,30], viral infections [31], mortality and graft loss in transplantation [32], highlighting its extensive role of B2M in the biology of an individual.

The comparison of the B2M protein sequences of different species indicated that B2M does not show strong sequence conservation among species. The human B2M protein shares 70% sequential identity with that of mice [33]. Furthermore, interestingly, no intraspecific genetic polymorphisms have been observed in human *B2M* [34], suggesting that changes in the B2M protein sequence could be undesirable to the host.

Pigs have not only long been an important domestic animal, but also recently emerged as a potential model for medical research, especially in immunity and xenotransplantation studies because of its suitable organ size and similarity to human biological system [35–38]. In view of this, a pig genome project was undertaken by the international pig genome consortium [39–41]. However, considering that 33.07% of the pig genome assembly includes the low-coverage and low-quality regions, it is still required to further improve the accuracy of the pig reference genome (Sscrofa10.2) [42].

The functional duplication of the *B2M* gene has not been reported in mammals. In this study, we identified the complete sequence duplication of the *B2M* gene in pigs, and validated the phenomenon and functionality. We also investigated the phylogenetic origins of the *B2M* duplication in related animal species. Our results confirmed that the functional duplication of *B2M* occurs only in pigs, and discussed the event of *B2M* duplication in the context of evolution, speciation, and adaptive changes in pigs.

## Methods

### *In silico* analysis of sequence data

Basic local alignment search tool (BLAST) analysis using pig *B2M* coding sequence (accession number, L13854.1) against the pig genome (Sscrofa10.2) revealed the presence of two genomic segments containing *B2M* in the genome. Based on the publicly available annotation of the pig genome assembly Sscrofa10.2 in the NCBI genome database [41], we performed a comparative analysis of the genetic structures of the duplication region among nine mammalian species described below. We found that the phylogenetically conserved *EIF3J* and *TRIM69* genes flanked the identified duplication in the pig chromosome 1 (GenBank accession number NW_003609204). The 358.5-kb sequence corresponding to the *EIF3J-TRIM69* interval was downloaded from the NCBI database with full features of the GenBank format from nine different species, including pigs (*Sus scrofa*, 358.5 kb, accession number NW_003609204: 388,903..738,903), mice (*Mus musculus*, 150.9 kb, NT_039207: 62,896,359..63,073,359), humans (*Homo sapiens*, 230.8 kb, NT_010194: 21,253,126..21,504,126), cats (*Felis catus*, 214.3 kb, NT_187881: 420,420..800,420), dogs (*Canis lupus familiaris*, 201.9 kb, NW_003726113: 11,200,900..11,438,900), horses (*Equus caballus*, 178.4 kb, NW_001867387: 77,431,718..77,620,718), sheep (*Ovis aries*, 213.8 kb, NW_004080170: 99,778,000..100,000,000), goats (*Capra hircus*, 211.5 kb, NW_005100768: 7,424,851..8,164,851), and cattle (*Bos taurus*, 214.3 kb, NT_187881: 420,420..800,420). Subsequently, these sequences were subjected to dot-plot analysis using the Gepard program (version 1.4) [43] to identify the relative positions and sizes of the duplicated blocks. Sequence alignments were performed using ClustalX embedded in the CLC Main Workbench (version 7.8.1) (CLCbio, Qiagen, Aarhus, Denmark). A multiple alignment for the syntenic comparison was carried out using the progressiveMauve program [44]. The analysis of the repetitive elements was carried out using the RepeatMasker Web Server with a sliding window of 10 kb [45]. For phylogenetic analysis, a sequence alignment with 11 full-length amino acid sequences of B2M from 10 species including human, mouse, cat, dog, horse, sheep, goat, cattle, pig and whale was generated using CLC Main Workbench software. Phylogenetic analysis was carried out using MEGA 6.0 software [46], and trees were constructed using Maximum Likelihood method based on Jones-Taylor-Thornton (JTT) model [47] with a bootstrap test of 1000 replicates.

### Cell culture

Cell lines, including HEK-293T (human embryo kidney, CRL-3216), PK13 (pig kidney of Hampshire breed, CRL-6489), and NIH3T3 (mouse embryo fibroblast, CRL-1658) were purchased from the American Type Culture Collection (ATCC). The cells were cultured in DMEM/high glucose media (Hyclone, UT, USA) supplemented with 10% FBS (Hyclone), 1% penicillin-streptomycin (Gibco, NY, USA), and 2 mM L-glutamine (Gibco), and 5% CO2 at 37°C.

### Preparation of DNA and RNA

To prepare genomic DNA, cells were trypsinized, collected, and subjected to DNA extraction. Briefly, cells were suspended in a lysis buffer [10 mM Tris-HCl (pH 8.0) and 0.1 M EDTA] containing 0.5% sodium dodecyl sulfate (SDS) and 20 μl of 20 mg/ml proteinase K (Promega, WI, USA) and incubated at 55°C for 6 h. The supernatant was purified with phenol/chloroform extraction, and the resulting DNA pellets were obtained by alcohol precipitation. The total RNA was extracted from cells using the Trizol reagent (Invitrogen, CA, USA). To extract total RNA from pig tissues, 1 ml of Trizol was added to approximately 100 mg of sample, and the tissues were homogenized. The purification step was conducted using RNeasy Mini Kits (Qiagen, Hilden, Germany) according to the manufacturer’s instructions. The quantification of nucleic acids was carried out using NanoDrop 1000 spectrophotometer (Nanodrop, DE, USA), and their integrity was checked by electrophoresis on 1% agarose gel in 1X Tris-acetate-EDTA (TAE) buffer. The gel was stained with ethidium bromide, and visualized under UV light.

### Genomic Polymerase Chain Reactions (PCRs)

To amplify the duplicated regions of *B2M* with sequence variations, the primers were designed for the conserved *B2M* intron-2 region (universal primer, UniblockAB-F) and the variable exon 4 (copy-specific primers, BlockA-R3 and BlockB-R), which can produce amplicons containing sequences from the partial *B2M* intron 2 to the downstream region of exon 4 from both copies of the *B2M* gene in the pig genome. The detailed information of the primers is presented in Table 1. Amplification reactions were performed in 50 μl containing 50 ng genomic DNA, 1 μM of each primer, 1.6 mM dNTPs, 1X PCR buffer (containing 2.5 mM MgCl_2_), and 2.5 U of LA Taq DNA Polymerase (TaKaRa, Kyoto, Japan) using a T3000 Thermocycler (Biometra, Goettingen, Germany). The cycling profile consisted of an initial denaturation at 94°C for 5 min, followed by 35 cycles of denaturation at 98°C for 10 s, annealing at 53°C for 20 s, and extension at 72°C for 2 min, and a final extension at 72°C for 10 min. For genomic PCRs with other primers, identical cycling conditions were used, except specific annealing temperatures used for each primer set (Tables 1 and 2). The semi-quantitative PCR for the *B2M* and *PATL2* genes was carried out to compare the band intensities of the amplicons for each *B2M* exon, exon 1 (gB2M-e1F and gB2M-i1R), exon 2 (gB2M-i1F and gB2M-e2R), and exon 3 (UniblockAB-F and gB2M-i3R), and *PATL2* exon 4 (gPATL2-i3F and gPATL2-i4R) to that of *GCG* exon 4 (gGCG-i3F and gGCG-e4R), a single copy gene. The PCR conditions were identical to those described above, except that the number of cycles was reduced to 23. The PCR products were analyzed by electrophoresis on 1% agarose gel in 1X TEA buffer.

**Table 1:** Primers used for semi-quantitative PCR and examination of the sequence variation between duplicated block A and B.

Targets	Template	Primer name	Sequence (5' - 3')	Annealing temprature (°C)	Product size
Block-A	genomic	UniblockAB-F	CCTAACAAGGCGCTCATGGTC	62	1724
		BlockA-R3	GGGCTGCTCCCCCGGCA		
Block-B	genomic	UniblockAB-F	CCTAACAAGGCGCTCATGGTC	62	1782
		BlockB-R	GAGGAGTTTCACTCTTGTCCGAG		
long-B2M	cDNA	B2M-F1	TTCArGTwTACTCACGyCAyCCA	53	281 (278 in pig)
		B2M-R	TyAswkGTCTCGATCCCA		
long-GAPDH	cDNA	GAPDH-F1	CATCACCATCTTCCAGGA	53	476
		GAPDH-R1	CCATGCCAGTGAGCTTC		

Note: Lower case letters for nucleotide sequences indicate the degenerated nucleotides.

**Table 2:** Primers used for quantitative PCR of the β2-microglobulin gene.

Targets	Template	Primer name	Sequence (5' - 3')	Annealing temprature (oC)	Product size
short-B2M	cDNA	B2M-F2	CAGCAAGGACTGGTCTTTCTA	53	133
		B2M-R	TyAswkGTCTCGATCCCA		
short-GAPDH	cDNA	GAPDH-F2	CCTGGCCAAGGTCATCCA	53	123
		GAPDH-R2	CGGCCATCACGCCACAG		
gB2M -exon 1	genomic	gB2M-i0F	GCCGAGCTCTCATTCCACC	60	150
		gB2M-i1R	GGCTGGTAGAAGAGGGAAGAGG		
gB2M-exon 2	genomic	gB2M-i1F	CTTTCCTTGATGTTCCTCAG	60	198
		gB2M-e2R	TAGAAAGACCAGTCCTTGCTG		
gB2M-exon 3	genomic	UniblockAB-F	CCTAACAAGGCGCTCATGGTC	60	257
		gB2M-i3R	CTGCAGACAGTGAGTGCCAG		
gPATL2-exon 4	genomic	gPATL2-i3F	CCTGGGTTTTCCTCCCTCC	60	210
		gPATL2-i4R	GTCAAGAGCAGACTGCATGTC		
gGCG-exon 4	genomic	gGCG-i3F	GCCACTGCTTATAGGTGAGAACC	60	206
		gGCG-e4R	GCCTTCCTCGGCCTTTCA		

Note: Lower case letters for nucleotide sequences indicate the degenerated nucleotides.

### cDNA synthesis and RT-PCR

cDNA synthesis was performed in a 25-μl reaction using 5 μg of the total RNA, 50 ng of oligo-(dT)15 and SuperScript III ReverseTranscriptase (Invitrogen, CA, USA) following manufacturer’s instructions. The universal primers, B2M-F1 and B2M-R, which are located at the beginning of the exon 2 and at the end of exons 2 and 3, respectively, were designed for the semi-quantitative amplification of the *B2M* transcripts from human, mouse, and pig cells against the most conserved region across species while having degeneration at a few nucleotides showing variations among species (Table 1, S1 Fig). For control, *GAPDH* (GAPDH-F1 and R1) was used (Table 1). Amplifications were performed in 20-μl reactions containing 1 μl synthesized cDNA, 0.5 μM of primers specific for each gene, 200 μM dNTPs, 1X PCR buffer [10 mM Tris (pH 8.3), 50 mM KCl, 1.5 mM MgCl], and 0.5 U of Super-Therm DNA polymerase (JMR Holdings, Kent, UK) using a T3000 Thermocycler (Biometra). The thermal cycling conditions consisted of an initial denaturation at 94°C for 3 min, 26 cycles of denaturation at 94°C for 20 s, annealing at 53°C for 20 s, and extension at 72°C for 45 s, followed by a final extension at 72°C for 5 min. The PCR products were analyzed by electrophoresis on 1% agarose gel in 1X Tris-acetate-EDTA (TAE) buffer.

### Direct sequencing

PCR products were gel-purified using QIAquick Gel Extraction Kit (Qiagen) following the manufacturer’s protocol. Direct sequencing reactions were carried out using ABI PRISM BigDye Terminator Cycle Sequencing Kit (Applied Biosystem, Foster City, CA, USA) using 2 μl of the eluted PCR products with the same primers as in PCR reactions (Table 1). The products were analyzed on an 3730XL automated DNA analyzer (Applied Biosystem).

### Real-time PCR

Primer sets used to estimate the expression levels of mRNA via real-time PCR are described in Table 2 and S1 Fig. Reactions to compare the expression levels of B2M and GAPDH were performed in 25-μl containing 1 μl of synthesized cDNA, 0.25 μM of species nonspecific *B2M* primers (B2M-F2 and B2M-R) or *GAPDH* (GAPDH-F2 and GAPDH-R2) together with 1X solution of Rotor-Gene TM SYBR R Green PCR kit (Qiagen) using CFX ConnectTM Real-Time System (Bio-Rad, CA, US). The thermal cycling conditions consisted of an initial denaturation at 94°C for 3 min, 40 cycles of denaturation at 94°C for 20 s, annealing at 53°C for 20 s, and extension at 72°C for 20 s, followed by a final extension at 72° for 5 min. The following primer sets were used to estimate the gene copy number from genomic DNA using real-time PCR: gB2M-i0F and gB2M-i1R for *B2M* exon 1, gB2M-i1F and gB2M-e2R for *B2M* exon 2, uniblockAB-F and gB2M-i3R for *B2M* exon 3, gPATL2-i3F and gPATL2-i4R for *PATL2* exon 4, and gGCG-i3F and gGCG-e4R for *GCG* exon 4. The PCR conditions were the same as with above-mentioned cDNA amplification, except 60°C annealing temperature. The results were evaluated four times for each amplicon. The PCR results were analyzed by Bio-Rad CFX Manager, version 3.1 (Bio-Rad). The 2^-ΔΔCt^ method was used for the relative quantification to determine the expression level or gene copy numbers [48]. The PCR efficiencies of real-time PCR primers for B2M expression of each species were calculated from standard curve of serial 10-fold dilution (4 dilutions) amplifications for each type of cDNA (S2A, S2B and S2C Fig). P-values were calculated using analysis of variance (ANOVA) and multiple comparisons of means were performed using Tukey’s test embedded in the basic R program (version 3.2.3).

### Expression constructs and transfection

A complete coding sequence of the *B2M* gene was amplified from the total RNA of PK13 pig kidney cells (ATCC, CRL-6489) using primers, EcoRI-Linker-B2M-F (5′-AAA GAA TTC CGA GTG GTG GTT CGG CTC CCC TCG TG GCCT-3′) and B2M-HIS-SalI-R (5′-AAA GTC GAC TTA GTG GTG ATG GTG ATG ATG GTG GTC TCG ATC CCA-3′) by RT-PCR. The amplification reactions were performed in 25-μl reaction volume containing 1 μl of synthesized cDNA, 0.5 μM each of primers, 200 μM dNTPs, 1X PCR buffer, and 0.5 U of Pyrobest DNA polymerase (Takara) using a T3000 Thermocycler (Biometra). The thermal cycling conditions included initial denaturation at 94°C for 3 min, 35 cycles of denaturation at 94°C for 20 s, annealing at 56°C for 20 s, and extension at 72°C for 45 s, followed by a final extension at 72°C for 5 min. The PCR products were gel-purified using QIAquick Gel Extraction Kit (Qiagen). The amplicon was ligated into the pCMV-HA-N vector (Clontech, CA, USA) to generate pCMV-HA-B2M-His construct after double digestion with *EcoR*I and *Sal*I restriction enzymes. The ligation products were transfected into DH10B cells (Invitrogen) using MicroPulser (Biorad, CA, USA). The transformed bacteria were plated on agar containing 50 μg/ml ampicillin. Five colonies were picked for each construct, and the plasmids were isolated from 3-ml cultures using Plasmid SV Miniprep Kit (GeneAll Biotechnology, Seoul, Korea). The absence of mutations in the constructs were confirmed by sequence analysis of the inserts using pCMV-HA-N-F (5′-CTC AGT GGA TGT TGC CTT-3′) and pCMV-HA-N-R (5′-AAA AAC CTC CCA CAC CTC-3′) as forward and reverse sequencing primers, respectively. The plasmids were transfected into the PK13 cells using Lipofectamine 2000 Kit (Thermo Fisher Scientific, MA, USA) according to the manufacture’s protocol.

### Immunocytochemistry

Lung fibroblasts, which express a low level of swine leukocyte antigen (SLA) class I [49], were obtained as a kind gift from Dr. Jin-Hoi Kim, Konkuk University. The cells were seeded on glass coverslips (Warner Instruments, CT, USA) in a 6-well plate at a density of 1.2 × 10^5^ cells/well. On day 2, approximately 80% confluent cells were transfected with expression constructs, pCMV-HA, pCMV-B2M, or pEGFP, as described above. After 24 h, cover slips with attached cells were washed with cold phosphate buffered saline (PBS), and fixed with 4% paraformaldehyde for 15 min at room temperature. Cells were washed with cold PBS before blocking with 5% bovine serum albumin (BSA) in PBS for 30 min at room temperature. The cells were incubated with rabbit monoclonal antibodies specific to HA tags (dilution 1:250, clone C29F4; Cell Signaling Technology, MA, USA) or mouse monoclonal antibodies specific to SLA class I heavy chains (dilution 1:200, clone JM1E3; AbD Serotec, OX, UK) for 2 h at room temperature. After rinsing with cold PBS, cells were incubated with the goat anti-mouse/anti-rabbit antibody (1:500) conjugated with Alexa 568 (Invitrogen) for 1 h at room temperature, and the coverslips were mounted with a drop of Vectashield (with DAPI) (Vector, CA, USA) on a microscope slide. The cells were visualized using a fluorescence microscope DP72 (Olympus, Tokyo, Japan).

### Fluorescence-activated cell sorting (FACS)

Lung fibroblasts transfected with expression constructs, pCMV-HA or pCMV-B2M, were collected after culturing for 24 h after transfection, fixed with 2% paraformaldehyde for 15 min at room temperature, and incubated with mouse monoclonal primary antibodies specific for SLA class I (1:250 dilution, clone JM1E3; AbD Serotec). Cells were washed, and subsequently incubated with goat anti-mouse secondary antibody (1:500) conjugated with Alexa 568 (Invitrogen) for 1 h on ice in dark. Cells were washed, resuspended in cold PBS, and analyzed using FACSCanto II flow cytometer and FACSDiva program version 6.1.3 (BDbiosciences, NJ, USA). The experiments were replicated twice. The pEGFP-transfected cells were used as control.

## Results

### Structural analysis of the 358.5-kb B2M flanking region in the current pig genome assembly

**Fig 1:**
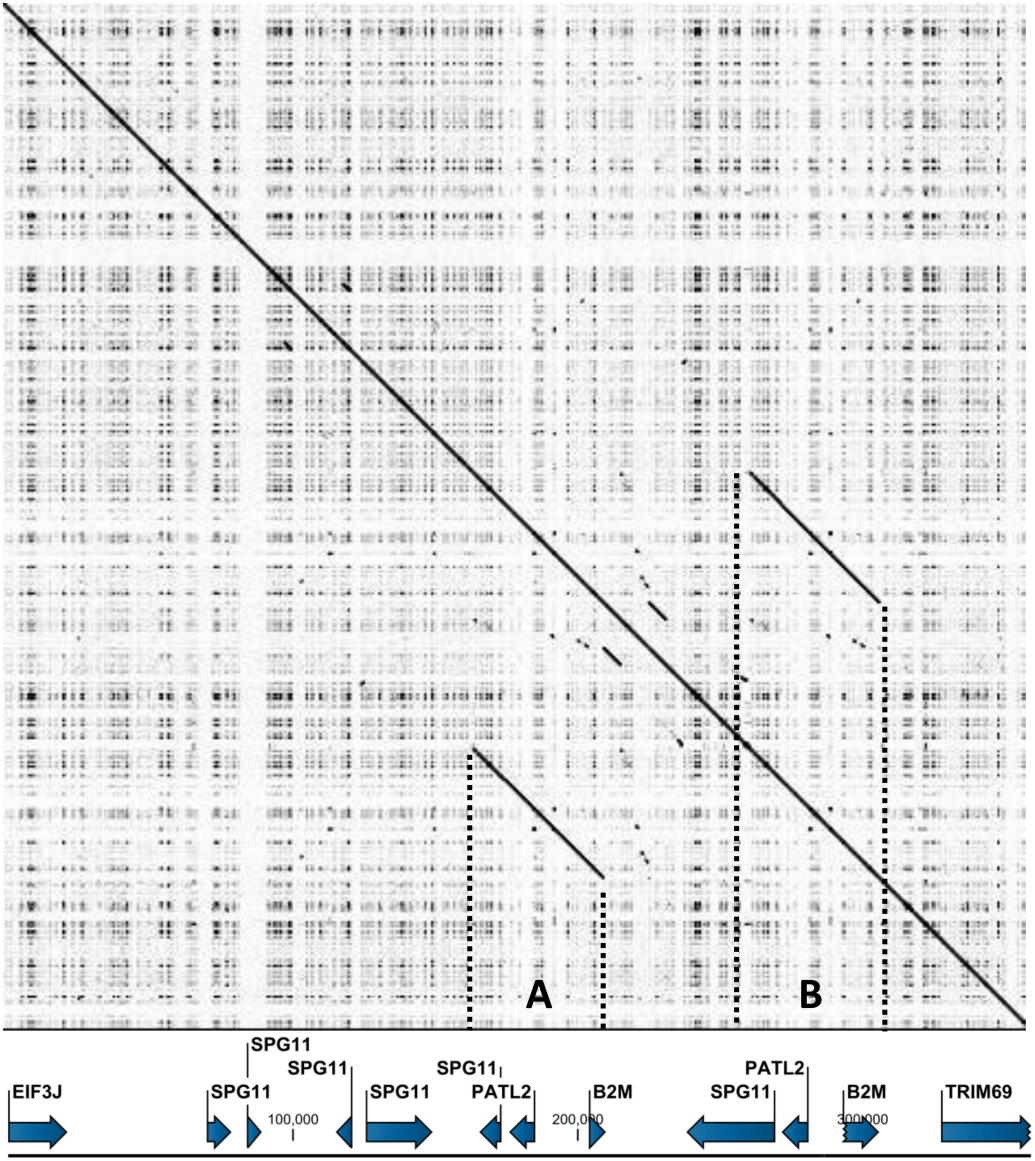
The self dotplot of the 358.5-kb *EIF3J-TRIM69* interval in pig chromosome 1. The sequence corresponding to accession number NW_003609204, position 388,903 to 738,903, was analyzed (Sscrofa10.2). The annotation is indicated at the bottom of the plot. A dot indicates the identical sequences of 10 bp. Two forward slope lines indicate duplicated blocks A and B, which are more than 99.5% identical in the ~45.5 kb segment containing the intact *B2M* gene. The region corresponding to each block is indicated by dotted vertical lines. The interspace between the two blocks on pig chromosome 1 is approximately 35 kb.

**Fig 2:**
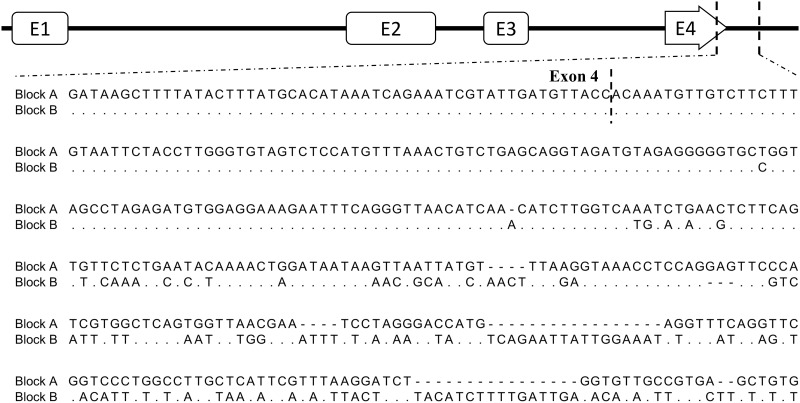
Comparison of the nucleotide sequences for the variable regions between the two copies of *B2M* genes. The exon and intron structures of *B2M* are shown on top. The alignment results of the two sequences obtained from the direct sequencing are shown. The 420-bp diversity region corresponding to the end of exon 4 between the two duplicated *B2M* genes is shown.

Examining the gene annotations of the current pig genome assembly (Sscrofa10.2), we detected an unexpected segmental duplication in the long arm of chromosome 1, including the *B2M* gene previously mapped to SSC1q17 without knowing the duplication [50]. For detailed characterization, a dot-plot analysis was performed on the selected region of the pig genome between the *EIF3J* and *TRIM69* genes (accession number, NW_003609204: 388,903–738,903). The results showed the presence of a segmental duplication with two identical blocks of 45.5 kb, which were separated by a gene fragment of 34.9 kb (Fig 1). In the current pig genome assembly, the 358.5-kb *EIF3J*-*TRIM69* interval comprises five genes, including *EIF3J*, *SPG11*, *PATL2*, *B2M*, and *TRIM69*. Moreover, this region was found to be highly evolutionarily conserved among other mammals. We named the two duplicated blocks as blocks A (NW_003609204.1, nucleotide position 553,595–599,255 and B (649,075–694,753), as indicated in Fig 1. The sequence alignment between the two duplicated blocks of 45.5 kb revealed the sequence identity of 99.4%, with major dissimilarity located in the exon 4 of the partial *SPG11* gene, downstream of the *PATL2* gene, and exon 4 of the *B2M* gene (Fig 2).

### Validation of the *B2M* duplication by genomic sequence analysis and real-time PCR

From the comparison of the 45.5-kb genomic sequences between the duplicated and original copies of the *B2M* gene containing blocks from the pig genome assembly Sscrofa10.2, we identified the regions showing nucleotide variations. Using the primers specific for the region displaying sequence differences between the two copies of *B2M*, which is located between the intron 2 and the downstream region of *B2M* exon 4, we amplified the *B2M* locus-specific amplicons of 1724 and 1782 bp for blocks A and B, respectively, from the genomic DNA prepared from a pig cell line PK13 (data not shown). The results from the direct sequencing of the two *B2M* amplicons showed similarity to the current pig genome assembly, and were mapped into two expected regions (Fig 2).

In addition, we carried out the semi-quantitative PCR against the entire coding region of the *B2M* (three amplicons from exon 1, 2 and 3) and *PATL2* (exon 4) genes, using the pig genomic DNA to estimate their copy numbers in the pig genome and *GCG* as a control for the single copy gene. The result showed that the band intensity of all three amplicons of *B2M* was relatively higher than that of the single copy control gene, even when considering the differences in fragment size (S3B Fig). However, the band intensity of *PATL2* amplicons did not support the duplication of *PATL2* in the pig genome. These observations were further confirmed by the results of the subsequent real-time PCR, by which the estimated copy numbers of the *B2M* exons 1, 2, and 3 were calculated to be 1.51, 1.36, and 1.97 times, respectively, as compared to *GCG*, and 2.12, 1.9 and 2.77 times, respectively, as compared to *PATL2* (P < 0.0001, S1 Table). The copy numbers of the B2M exons 1 and 2 were slightly lower than that of the exon 3 because of the difference in their product sizes and amplification efficiency compared to the control GCG; however, the combined results from all three exons clearly showed the presence of an extra copy of *B2M*. Considering that no genetic polymorphisms have been reported in the human *B2M* gene, and that we also did not observe any polymorphisms in the pig B2M gene from our analysis using public databases and sequencing results of the *B2M* exon 2, the largest exon, from one animal each of the seven pig breeds (data not shown), it was less likely that the detected variations were allelic. Therefore, we confirmed the genomic duplication of *B2M* in pigs both by analyzing the sequence variation between the two copies of *B2M* and by estimating the copy number using the real-time PCR. However, our results indicated that the duplication of *PATL2* in the genome assembly Sscrofa10.2 was due to the assembly errors.

### Functional validation of *B2M* duplication in pigs by comparing the levels of *B2M* mRNA among humans, mice, and pigs

**Fig 3:**
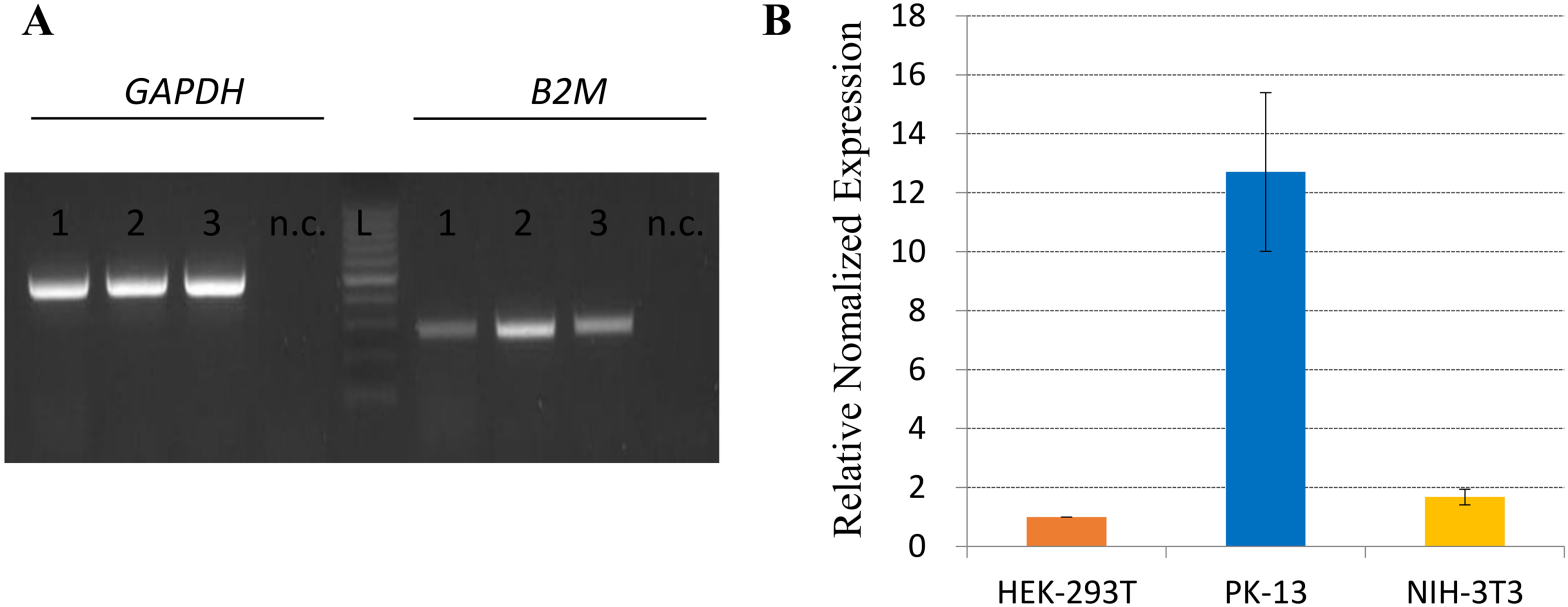
Comparison of the expression levels of the human, pig, and mouse *B2M* transcripts using the semi-quantitative RT-PCR (A) and real-time PCR (B). The *GAPDH* gene was used as a single copy gene control. In real-time PCR, each reaction was repeated four times and the relative expression level of each sample to HEK-293T cells (human) was indicated in Y-axis (ΔΔCt method). Cell lines, HEK-293T (human), PK13 (pig) and NIH-3T3 (mouse) were showed in (A) as 1, 2 and 3, respectively. n.c, negative control, L, DNA ladder.

To evaluate whether the expression level of *B2M* mRNA in pigs was relatively higher than that of the other species with a single copy of *B2M*, we amplified the *B2M* gene by both semi-quantitative PCR and real-time PCR, using cDNA from the human, mice, and pig cell lines (Fig 3, S3A Fig, S2 Table). In semi-quantitative PCR, the band intensity was significantly higher in pigs than the other animals. Furthermore, the expression level of *B2M* mRNA in pigs was significantly different from humans and mice as shown by the real-time PCR (p < 0.001). Surprisingly, the *B2M* expression level in pigs was 12.71 and 7.56 times (2^-ΔΔCt^ values) higher than humans and mice, respectively, which could not be explained only by the difference in their gene copy numbers. This result indicates that there could be an additional mechanism for the transcriptional control of the *B2M* gene in pigs because of the biological importance and evolutionary need of the *B2M* gene for this species. The higher level of *B2M* expression was also confirmed on pig tissues including the small intestine and kidney (S4 Fig).

The amplification efficiency of the *B2M* gene among different species might be different because of the bias from the sequence variations among different species. However, we attempted to overcome this bias by using a pair of universal primers with a few degenerate nucleotides for the amplification of *B2M* from different species after carefully considering the patterns of nucleotide variations. Our results suggested that both gene duplication and transcriptional regulation contribute to the increased levels of *B2M* transcripts in pig cells, which might lead to a beneficial effect on the immune system of pigs. Because of the complete sequence identity in the exonal region between ancestral and duplicated copies of *B2M*, differentiation of expression level between the two copies was not possible. We assumed that the expression of both copies of *B2M* contribute to higher *B2M* expression in pigs comparing to the species with a single B2M such as humans and mice.

### Over-expression of HA-tagged *B2M* mRNA did not correlate with enhanced expression of SLA class I complexes on the cell surface

**Fig 4:**
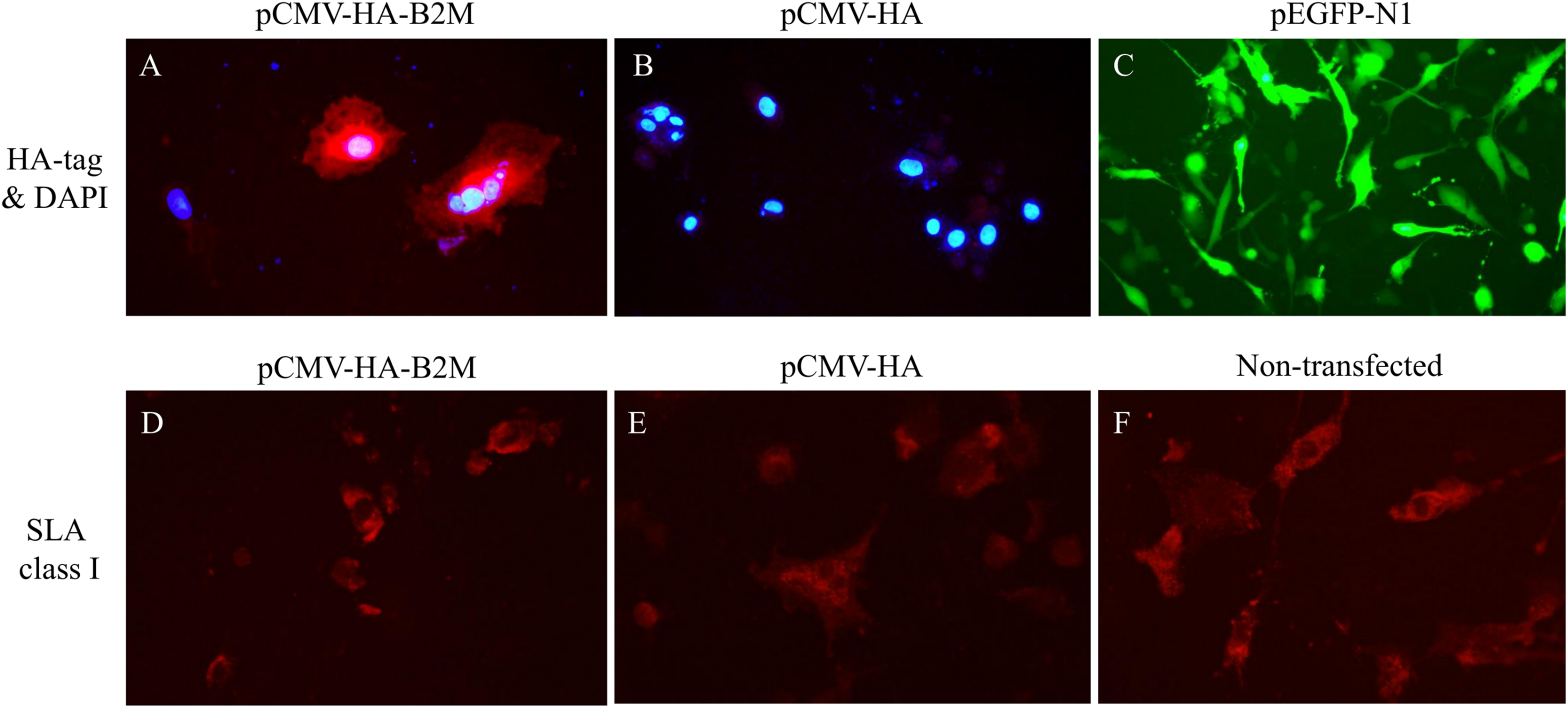
Immunohistochemical analysis of the B2M and SLA class I expression in cells overexpressing B2M. The names of the transfected constructs are shown on top: pCMV-HA-B2M for the expression of HA-tagged B2M, pCMV-HA for the HA tag without B2M, and pEGFP-N1 for EGFP expression. The primary antibodies used for the analysis are shown on the left. A and B. Cells were stained with the HA tag-specific antibody. The cell nuclei were visualized using 4′,6-diamidino-2-phenylindole (DAPI; blue). C. pEGFP (green) plasmid was used to evaluate the transfection efficiency. D, E, and F. SLA class I heavy chains were stained with pan SLA class I-specific antibodies. The signals of primary antibodies were detected by Alexa 568-conjugated secondary antibody (red) in all cases.

An increase in the levels of B2M proteins could affect the consequences of immune reactions in response to pathogenic infections. To understand the relationship between the expression level of HA-tagged *B2M* mRNA and the number of MHC proteins presented on the cell surface, we analyzed the changes in the HA signal intensity on the cell surface of the cells transfected with HA-tagged *B2M*. Although the formation of a functional MHC class I complex between 6xHis-tagged B2M and HLA class I genes has been previously reported [51], the complex formation between the HA-tagged recombinant B2M and MHC class I molecules has not been reported yet. The immunocytochemical analysis using HA specific antibodies showed clear signals on the surface of transfected cells, indicating that the HA-tagged recombinant B2M also properly forms complexes with SLA class I molecules (S5 Fig). Therefore, we estimated changes in the expression of MHC molecules on the surface of primary lung fibroblast cells transfected with HA-tagged B2M by measuring the level of both HA and SLA class I signals using antibodies specific for HA and SLA class I heavy chains, respectively (Fig 4). However, no significant change was observed in the number of SLA class I molecules presented on the surface of cells overexpressing HA-tagged B2M (Fig 4b). This observation was supported by the FACS results, which showed almost no changes in the SLA class I expression on the surface of cells transfected with and without the B2M coding sequence. In fact, the cells transfected with pCMV-HA-B2M showed slightly lower signals of SLA class I molecules (79.9 and 74.1%, obtained from two experiments) than the cells transfected with pCMV-HA (80 and 80.1%) (S6 Fig). This result suggested that the HA tag fused to B2M might negatively influence the efficient formation of a B2M-SLA I complex. However, further studies are required to address this issue.

### Comparisons of genetic structures for the *EIF3J-TRIM69* syntenic region among different species

**Fig 5:**
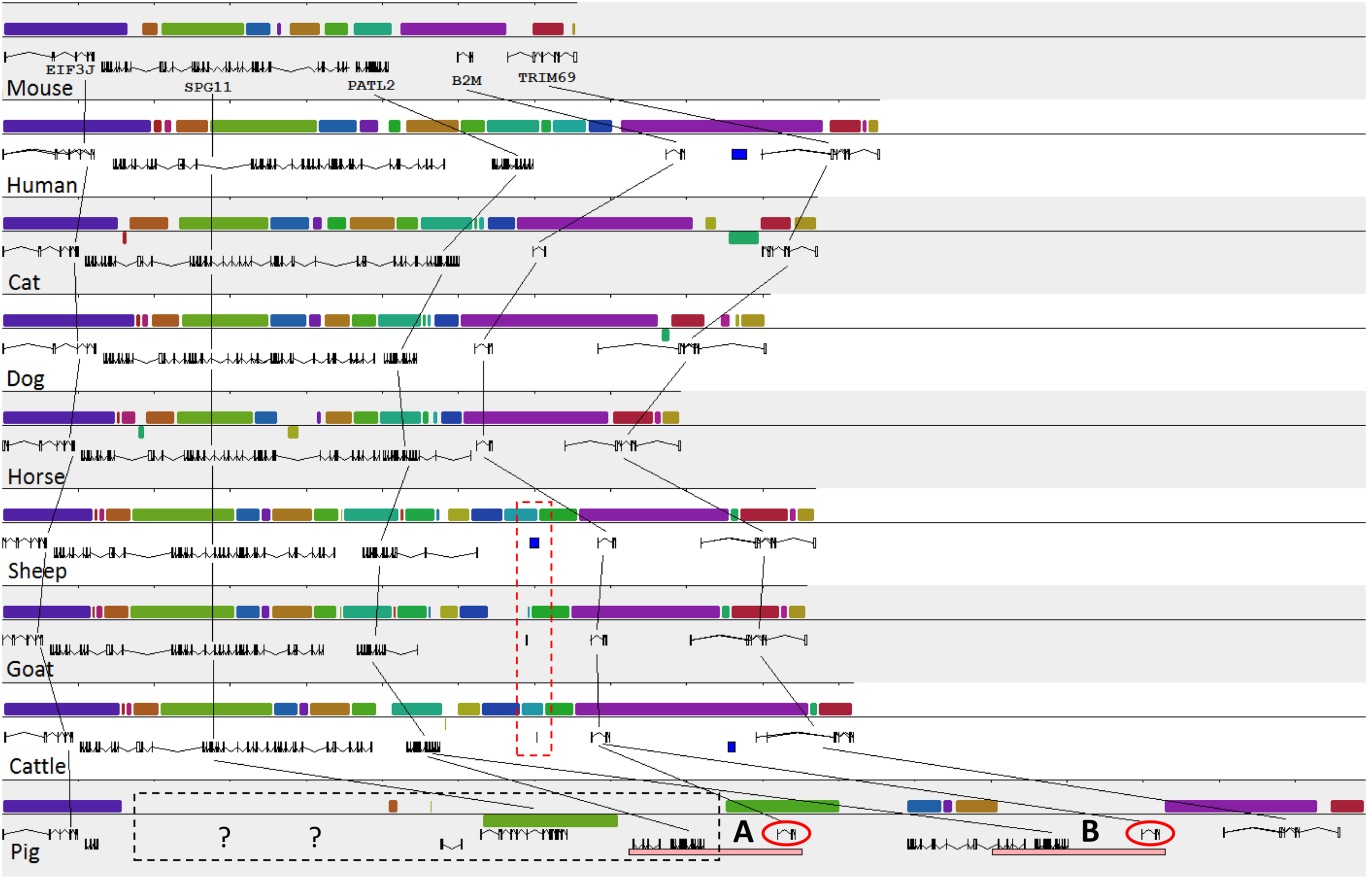
Comparison of the genetic structure of the *EIF3J-TRIM69* interval among nine mammals. The same colors indicate the corresponding elements in the genomes indicated by the lines. The exon and intron structures of the genes are shown under each element. The structure of the pig genome was based on the pig genome assembly Sscrofa10.2. The segmental duplications A and B in pigs are indicated by pink bars. The red dashed box indicates the partial sequences (~800 bp) of the *B2M* gene remnants in the cetartiodactyl species. The black dashed box with question marks indicates the low-confidence region from the current pig genome assembly (Sscrofa10.2), which shows discrepancy with our experimental data. The two *B2M* genes of pigs are indicated by the red circles.

To understand the evolutionary aspects of *B2M* duplication, we compared the annotations of the *EIF3J*-*TRIM69* interval from the publicly available genome information among diverse species including the closely related artiodactyls, such as cattle, goats, and sheep, and more distantly related horses, dogs, cats, mice, and humans together with pigs. The comparison showed that the genetic structure of *EIF3J*-*TRIM69* interval was highly conserved among different species, except for the slight difference in their sizes (Fig 5). The conserved segments in all species, except pigs, were in the same order without any significant rearrangements. Surprisingly, the size of the *EIF3J*-*TRIM69* interval was greatly expanded in pigs. Our real-time PCR results for the copy number estimation of *PATL2* showed conflict with its structure in the current pig genome assembly (S3B and S4C Figs). In addition, we were unable to validate the interruption of *SPG11* in pigs because the specific amplicons were amplified from reverse transcription (RT) PCR using primers specific to the break point of *SPG11* gene, including the intervals of exons 15 and 17, and 22 and 23, which should not be amplified if the gene configuration in the current pig genome assembly is correct (data not shown). Therefore, our experimental validation showed inconsistency between the information in the current pig genome assembly and our experimental data.

### Occurrence of *B2M* duplication in the cetartiodactyl lineage and intactness only in pigs

To assess the evolutionary history of *B2M* duplication event in animal species, we analyzed the presence of sequences corresponding to the *B2M* gene or the remnant in the syntenic region from the *EIF3J*-*TRIM69* interval from each of the nine other species using BLAST. Interestingly, we found the additional presence of partial sequences of exons 3 to 4 of *B2M* in sheep, goats, and cattle. The size and level of sequence similarity varied from 1.2 kb (99% identity) in sheep to 0.3 kb (99%) in goat and 1 kb (100%) in cattle. In addition, we identified the partial duplication of a 943-bp fragment corresponding to a region from the middle of intron 3 to the end of exon 4 of *B2M* from the Scaffold 142 (Oorc_1.1, ID: NW_004438556.1) of the genome sequence of a killer whale (*Orcinus orca*). More interestingly, we found a retro-transposition event containing a complete protein coding region of *B2M* on Scaffold 53 (Oorc_1.1, ID: NW_004438467) from the genome of *O*. *orca*. The retrogene sequence showed high similarity (87.3% identical) to the coding sequence of whale *B2M*, but lacked two amino acids (S7 Fig). This retrogene cannot be an artifact because we also found the same retrogene from the sperm whale genome (Physeter_macrocephalus-2.0.2 Scaffold 4325, ID: NW_006717073.1). However, the gene is possibly nonfunctional because we were not able to detect the corresponding sequence in the available gene expression data of the *O*. *orca* from the NCBI RNA RefSeq database.

**Fig 6:**
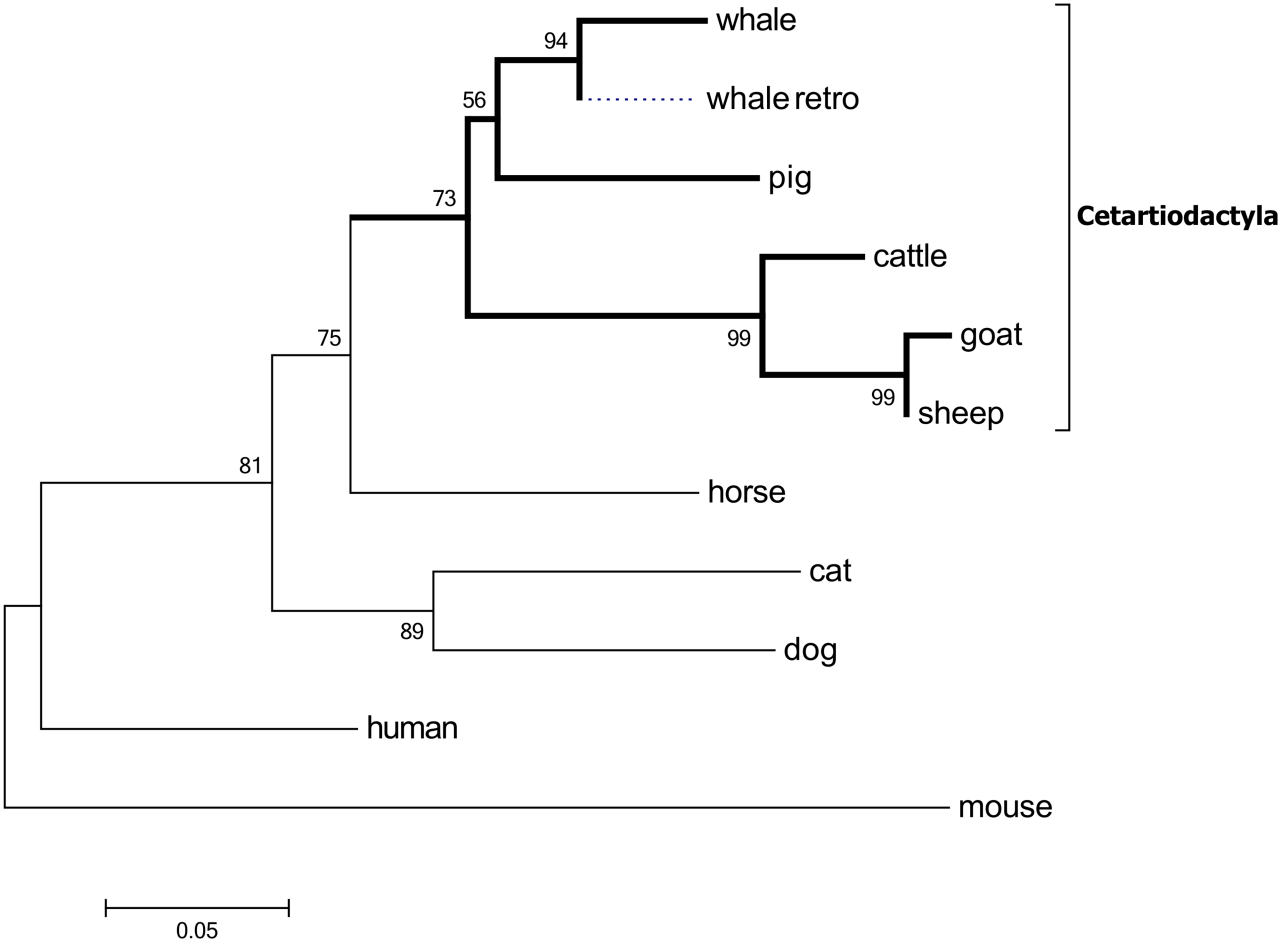
The phylogenetic analysis using the amino acid sequences of the *B2M* gene among ten mammalian species including whale (intact, XM004281255; retrogene, NW004438467), pig (L13854), cattle (BC118352), goat (XM013967395), sheep (NM001009284), horse (NM001082502), cat (NM001009876), dog (NM001284479), human (NM004048) and mouse (NM009735). Unrooted phylogenetic trees were constructed from 116–125 amino acids in length, from translated *B2M* of ten species using the maximum likelihood method and the representative tree was shown. The numbers on the nodes indicate the bootstrap values above 50% (n = 1000). Whale_retro indicates the retrotransposon copy of the *B2M* gene in whale. The node of pig represents for both copies of B2M with the identical amino acid sequence.

This observation suggests that the duplication event of *B2M* is common in cetartiodactyl species. However, we were unable to reconstruct the detailed structure of the *EIF3J*-*TRIM69* interval because of the limited information of the whale genome. The locations of the partially duplicated *B2M* sequences for the corresponding species, except for whale, are indicated in Fig 5. The presence of the partially duplicated sequence of *B2M* was not observed in species out of cetartiodactyla such as mice, humans, cats, dogs, and horses. This is in agreement with the phylogenetic relationship of the species constructed by amino acid sequences of the *B2M* gene, in which artiodactyl species formed a distinct cluster from the remaining species (Fig 6).

## Discussion

B2M is an essential protein with diverse biological roles, and therefore the genetic variations or mutations of the gene could result in biologically significant differences in the related phenotypes. In this study, we confirmed the presence of the double dose of the intact *B2M* gene in pigs with the identical coding sequence and that the remnant of *B2M* duplication is present in all analyzed cetartiodactyl species.

We were unable to confirm the effect of the *B2M* gene duplication on the level of protein expression and compare it among different species because of the unavailability of the “special” antibody, which is equally reactive to the *B2M* of different species. However, our results showed that the over expression of HA-tagged B2M might differ from the endogenous B2M without tags (Fig 4, S5 Fig), preventing a clear conclusion on this. Although we were not able to observe an increase in the cell surface expression of SLA class I molecules in the cells overexpressing HA-tagged B2M, it was possible that the actual expression level of SLA class I molecules from the two copies of B2M genes in pig cells was higher than the other species with a single copy of *B2M*.

In addition to the possible structural alteration of B2M by the addition of HA tags, the expression of MHC class I can also be regulated through the mechanism of MHC class I assembly and translocation to the cell surface. Besides B2M, the transporter associated with antigen processing (TAP), tapasin, the endoplasmic reticulum (ER) oxido-reductase ERp57, protein disulfide isomerase (PDI), the lectin chaperones, calnexin and calreticulin, and the ER aminopeptidase (ERAP) play important roles in the multilevel antigen presentation pathway machinery and have an effect on the expression level of SLA class I molecules [52]. Moreover, the upregulation of a single component can enhance MHC class I expression as in the case of ERAP1 protein [53]. Therefore, the double dose of endogenous B2M is likely to enhance the capacity of the pig immune system.

**Fig 7:**
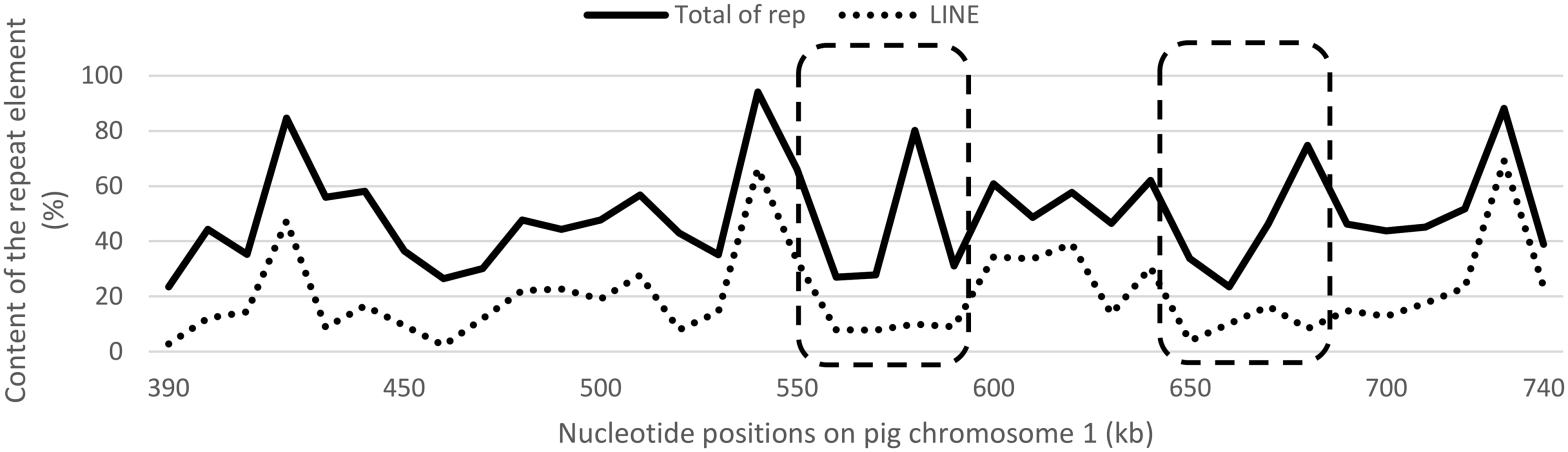
Distribution of the total repetitive elements (continuous line) and LINE (dotted line) contents within the *EIF3J*-*TRIM69* interval in pigs. The content of the repetitive elements was analyzed using RepeatMasker for 10 kb word count. The dashed boxes indicate the ~45.5-kb duplication regions.

Several mechanisms have been suggested for the chromosomal rearrangements. Largely, the outcomes of genetic recombination might sometimes lead to segment duplication [54–57]. Non-homologous end joining (NHEJ) and microhomology-mediated end joining (MMEJ) can lead to DNA recombination by double-strand break repair, which requires very short or no sequence homology [58,59]. In contrast, the non-allelic homologous recombination (NAHR) is involved in generating the recurrent copy-number variations through homologous recombination between repeated sequences [60]. We examined the composition of the repeat elements in the *EIF3J-TRIM69* interval (Fig 7), and found that the short-length repeat elements, such as short interspersed nuclear element (SINE) and *Alu* repeats, showed negligible presence (data not shown). However, the density of long interspersed nuclear elements (LINE) located at the edges of duplicated blocks was 2 to 3-fold higher (39 to 66%) than the average (20.12%), suggesting its role in the duplication event; however, the assembly information for this region needs further improvement. This phenomenon was consistent with the mouse chromosomal architecture in which a high content of LINEs and long terminal repeats (LTRs) were present within the boundaries of large duplication clusters [61,62]. Moreover, it was reported that the cetartiodactyl-specific evolutionary breakpoint regions (EBRs) are enriched for the LINE elements [41,63]. This suggested that the *B2M* segmental duplication might occur through the participation of repeat elements, especially LINEs.

In the early evolution of vertebrates (gnathostomata), the *B2M* gene was assumed to have translocated from its original location within the *MHC* region to a diverse chromosome location among vertebrate species by the activation of tandem duplication [64]. In zebrafish, the presence of an additional, although nonfunctional, *B2M* locus with approximately 60% amino acid similarity to its paralogue is consistent with the hypothesis of the recent genome-wide duplication in teleost fish [64]. Therefore, the B2M duplication in zebrafish differs from that in cetartiodactyla.

Among the cetartiodactyl species analyzed in this study, only pigs retained the completely duplicated *B2M* gene with an almost identical sequence, including the promoter and gene body regions. The presence of the remnant partial sequences of the duplicated *B2M* next to the intact version in the other cetartiodactyl species suggests that the duplication event of the *B2M* block occurred in the ancestor of cetartiodactyls. Subsequently, the vertical evolution caused the divergence of *B2M* shown in different species as well as the inactivation of an extra copy of the *B2M* in the other artiodactyls.

The increased dosage of B2M in pigs is intriguing because of the possible presence of a mechanism, which maintained and protected the duplicated copy from the pathways of neofunctionalization, subfunctionalization, or loss of function in this species. Changes in the B2M-related biological processes or behaviors of pig ancestors for adapting to environments might increase the need for maintaining an extra copy of B2M, and prevented the accumulation of variations in the duplicated paralogue.

In mammals, in addition to the odor receptor gene families [65], immune genes, especially *MHC* genes, show an extreme expansion across species for adaptation to pathogenic environments [11,13]. Recent analysis revealed that pigs have five classical (class Ia: *SLA-1*, *-2*, *-3*, *-5*, and *-12*) and four non-classical (class Ib: *SLA-6*, *-7*, *-8*, and -*11*) biologically functional SLA-I genes [41]. In humans, there are three class Ia genes (*HLA-A*, *-B*, and *-C*) and three class Ib genes (*HLA-E*, *-F*, and *-G*) [66–68]. Although the functions of diverse *MHC* genes in pigs are still not clear, a large number of heavy chains require relatively higher levels of B2M, to perform their realistic roles.

B2M also couples with other molecules to perform various functions. Similar in structure to the MHC class I molecules, the neonatal Fc receptor (FcRn) was reported to be associated with B2M for transporting IgG from the colostrum through the gut epithelium into the bloodstream of the neonate for short-term passive immunity [69–72]. The increased dose of B2M might benefit the immune protection of piglets. Melanoma is one of the most fatal skin cancers characterized by its aggressiveness and therapeutic resistivity [73]. One of the well-known pathways involves the reduction of antitumor response of the tumor-specific T cells through the alteration of MHC class I presentation on the malignant cells [74]. Studies have reported that one of the major causes of melanoma includes the loss-of-function mutations of B2M [75–77]. In several breeds of pigs including, Libechov minipig and Sinclair, the complete regression of melanoma has been reported [78–80]. It is tempting to hypothesize the association between *B2M* duplication and melanoma regression in pigs; however, we do not have any direct evidence to support this hypothesis. Furthermore, it has been reported that the pig chromosome 1 contains quantitative trait loci (QTL) for cutaneous melanoma, which were mapped near the *B2M* gene in the melanoblastoma-bearing Libechov minipig swine model [81].

## Conclusion

We experimentally confirmed that the *B2M* gene is duplicated and remains functional in the chromosome 1 of pig genome. However, in the other cetartiodactyls analyzed in this study, the partial B2M duplication was observed. Changes in the B2M-related biological processes or behaviors of pig ancestors for adapting to environments might increase the need for maintaining an increased dose of *B2M*, and prevent the accumulation of variations even in the duplicated paralogue. In addition to serving as the light chain of MHC class I molecules, B2M plays diverse roles in the immune system of pigs; thus, the double dose of B2M could provide the benefits of increased fitness to pigs.

## Supporting information

S1 Table Result of the estimated Ct values to calculate the B2M and PATL2 gene dosage in the pig genome using real-time PCR. (PDF)

S2 Table Result of the estimated Ct values to calculate the expression level of *B2M* from HEK-293T (human), PK-13 (pig), and NIH-3T3 (mouse) using real-time PCR. (PDF)

S3 Table Ct value of real-time PCR of *B2M* expression of HEK-293T (human), NIH-3T3 (mouse) and pig tissues. (PDF)

S1 Fig Primer positions for *B2M* amplification by semi-quantitative reverse transcription (RT) and real-time PCR to validate the B2M expression in humans, pigs, and mice. The cDNA sequence of human *B2M* was used as a reference sequence. The primer binding site were indicated in the box. The identical nucleotides are shown in dots.
(PDF)

S2 Fig Standard curve of B2M amplification of HEK-293T (A), PK-13 (B) and NIH-3T3 (C) cell line. Amplification efficiencies (E) were calculated automatically and showed in lower part.
(PDF)

S3 Fig A. The results of the reverse transcription (RT)-PCR using the same primers used for the real-time PCR to estimate the levels of *B2M* expression in cell lines, HEK-293T (human), PK13 (pig) and NIH-3T3 (mouse) which were showed in (A) as 1, 2 and 3, respectively. n.c, negative control, L, DNA ladder. B. Result of the semi-quantitative PCR (23 cycles) to estimate the copy number of the *B2M* and *PATL2* genes in the pig genome using the pig genomic DNA. The band intensities of the *B2M* exons are stronger than those of the *GCG* and *PATL2* genes. Amplicons specific for the GCG exon 4, *PATL2* exon 4, and *B2M* exons 1, 2, and 3 are shown. C. Results of the real-time PCR using the primers in B. E1, E2, and E3 indicate exons 1, 2, and 3. ΔCt indicates the difference in the amounts of amplicons between single copy control gene, *GCG*, and *B2M*. *PATL2* was used as a house keeping gene for comparison.
(PDF)

S4 Fig B2M expression comparison between human and mouse cell line to pig small intestine and kidney. The *GAPDH* gene was used as a single copy gene control. Each eaction was repeated three times and the relative normalized expression level of each sample to HEK-293T cells (human) was indicated in Y-axis (ΔΔCt method).
(PDF)

S5 Fig Result of the immunocytochemical analysis of the HA-tagged-B2M transfected PK13 cells. The signals, which indicate the expression of HA-B2M recombinant fusion protein (red), were observed in cytoplasm and on cell surface. The nuclei of cells (blue) were stained with 4′,6-diamidino-2-phenylindole (DAPI).
(PDF)

S6 Fig The comparison of the expression levels of the SLA class I molecules between the B2M-transfected and non-transfected primary lung fibroblasts using fluorescence-activated cell sorting (FACS). The expression construct, HA-tagged-B2M (pCMV-HA-B2M), HA tag only (pCMV-HA), and EGFP (pEGFP-N1) were transfected into cells. Pig SLA class I-specific antibodies and Alexa 568-conjugated anti-mouse IgG antibodies were used as primary and secondary antibodies, respectively. EGFP (pEGGP-N1) was used to evaluate the transfection efficiency (green GFP detected). Non-transfected cells were used as control. The analysis was performed twice for each construct.
(PDF)

S7 Fig Results of the sequence alignment of eleven *B2M* sequences, 116–125 amino acids in length, from ten species. The grey box indicates the deleted amino acid in the retrotransposon sequence. Whale_retro indicates the retrotransposon copy of the *B2M* gene in whale. NCBI accession number: whale, XM004281255; whale retrotransposon, NW004438467; pig, L13854; cattle, BC118352; goat, XM013967395; sheep, NM001009284; horse, NM001082502; cat, NM001009876; dog, NM001284479; human, NM004048; mouse, NM009735.
(PDF)
