# Supplementary material for: β2-microglobulin gene duplication in cetartiodactyla remains intact only in pigs and possibly confers selective advantage to the species
Source: PLoS One. 2017 Aug 16;12(8):e0182322. doi: 10.1371/journal.pone.0182322 (PMC5558954; doi:10.1371/journal.pone.0182322)
Supplement: S1 Table — (PDF) [file pone.0182322.s001.pdf]

Trial	*GCG*	*B2M* exon 1	*B2M* exon 2	*B2M* exon 3	*PATL2*
1	20.06	19.55	19.65	19.09	20.55
2	20.15	19.52	19.69	19.32	20.66
3	20.21	19.6	19.78	19.16	20.7
4	20.28	19.65	19.81	19.21	20.75
Mean	20.175	19.58	19.7325	19.195	20.665
