# Supplementary material for: β2-microglobulin gene duplication in cetartiodactyla remains intact only in pigs and possibly confers selective advantage to the species
Source: PLoS One. 2017 Aug 16;12(8):e0182322. doi: 10.1371/journal.pone.0182322 (PMC5558954; doi:10.1371/journal.pone.0182322)
Supplement: S2 Table — (PDF) [file pone.0182322.s002.pdf]

Trial	HEK-293T		PK-13		NIH-3T3	
	B2M	*GAPDH*	*B2M*	*GAPDH*	*B2M*	*GAPDH*
1	17.25	14.06	13.63	14.41	15.5	12.79
2	17.26	14	14.03	14.36	15.56	13.23
3	16.69	14.08	13.57	14.34	15.17	13.35
4	16.64	14.31	13.66	14.59	15.09	13.47
Mean	16.96	14.1125	13.7225	14.425	15.33	13.21
