# Supplementary material for: β2-microglobulin gene duplication in cetartiodactyla remains intact only in pigs and possibly confers selective advantage to the species
Source: PLoS One. 2017 Aug 16;12(8):e0182322. doi: 10.1371/journal.pone.0182322 (PMC5558954; doi:10.1371/journal.pone.0182322)
Supplement: S3 Table — (PDF) [file pone.0182322.s003.pdf]

Trial	Human		Mouse		Pig tissues			
					Small intestine		Kidney	
	B2M	***GAPDH***	***B2M***	***GAPDH***	***B2M***	***GAPDH***	***B2M***	***GAPDH***
1	21.12	21.53	21.86	21.30	18.17	20.59	16.86	20.08
2	21.03	21.64	21.84	21.48	18.08	20.44	17.10	20.43
3	21.07	21.47	21.68	21.27	18.01	20.52	16.90	19.96
Mean	21.07	21.55	21.79	21.35	18.09	20.52	16.95	20.16
