# Supplementary figures and images for: β2-microglobulin gene duplication in cetartiodactyla remains intact only in pigs and possibly confers selective advantage to the species
Source: PLoS One. 2017 Aug 16;12(8):e0182322. doi: 10.1371/journal.pone.0182322 (PMC5558954; doi:10.1371/journal.pone.0182322)

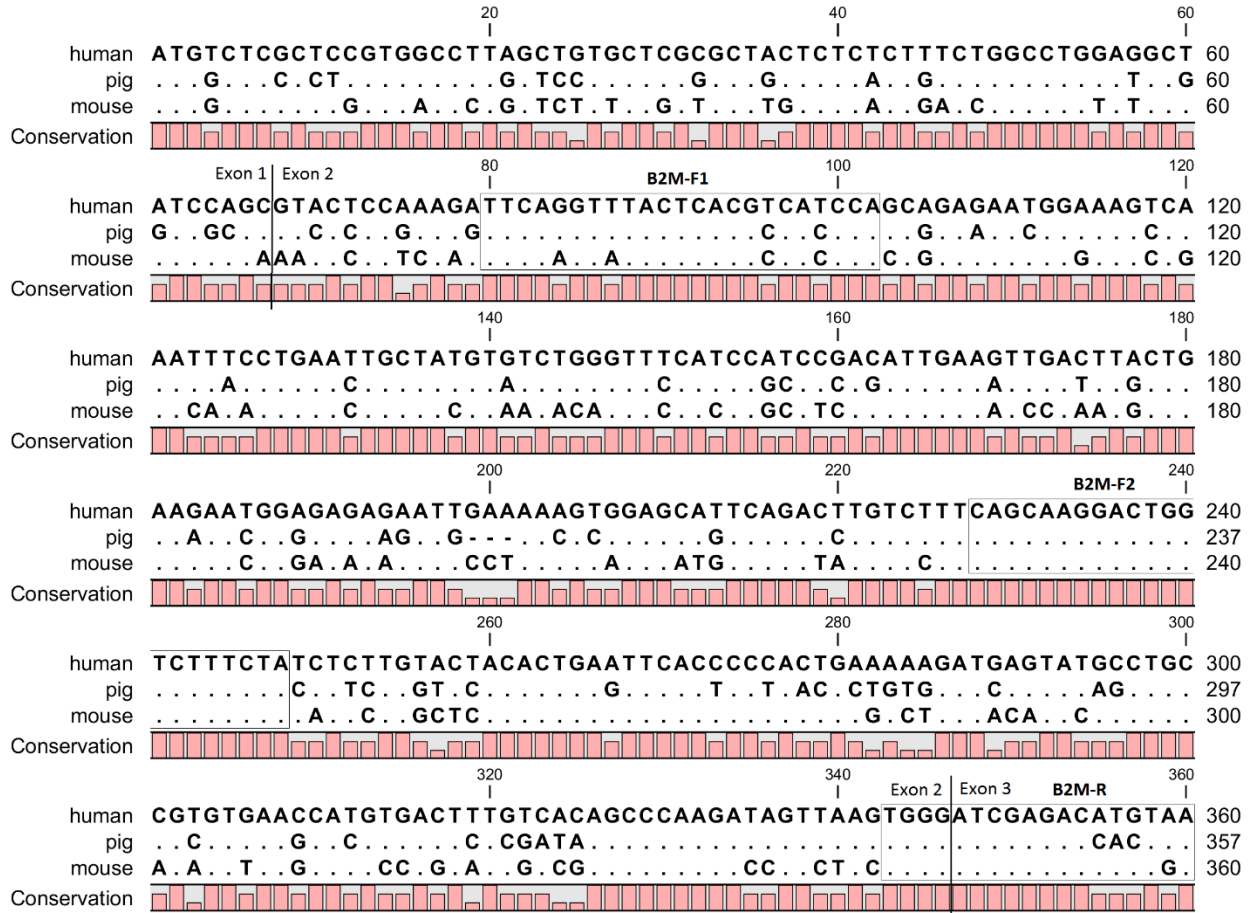

Supplement: S1 Fig — The cDNA sequence of human B2M was used as a reference sequence. The primer binding site were indicated in the box. The identical nucleotides are shown in dots. (PDF) [file pone.0182322.s004.pdf]

A

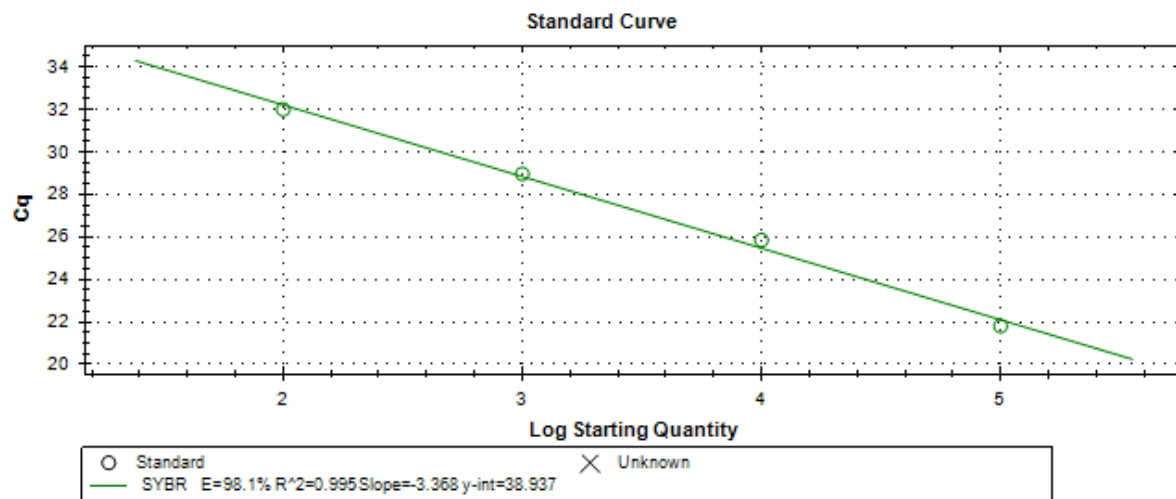

B

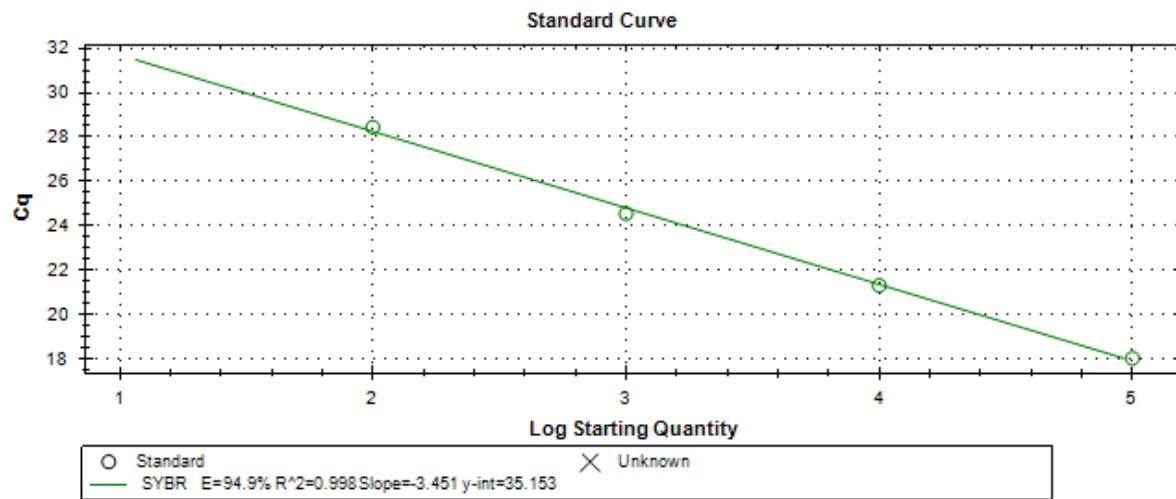

C

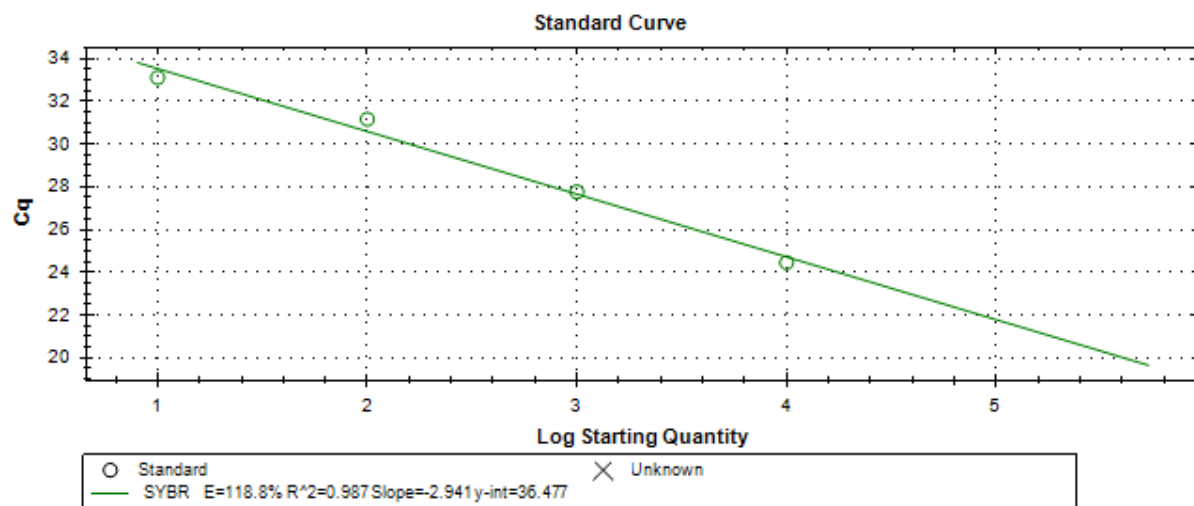

Supplement: S2 Fig — Amplification efficiencies (E) were calculated automatically and showed in lower part. (PDF) [file pone.0182322.s005.pdf]

**A**

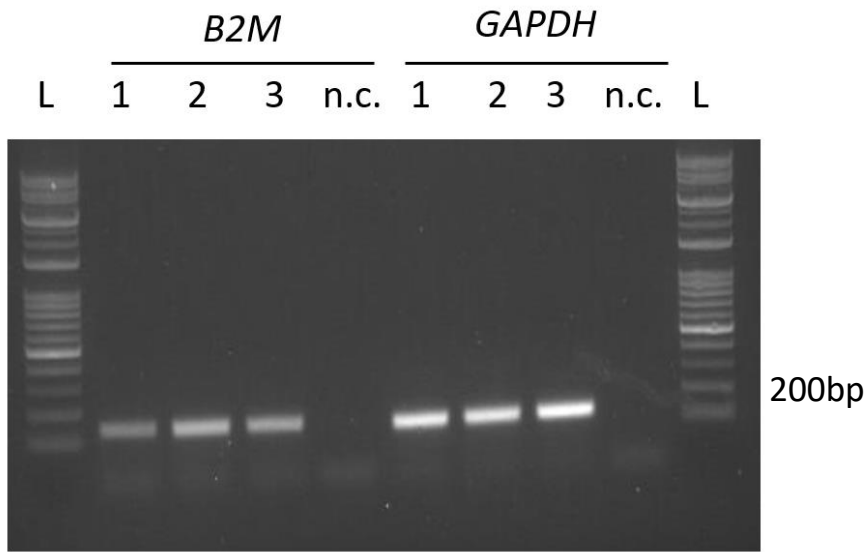

**B**

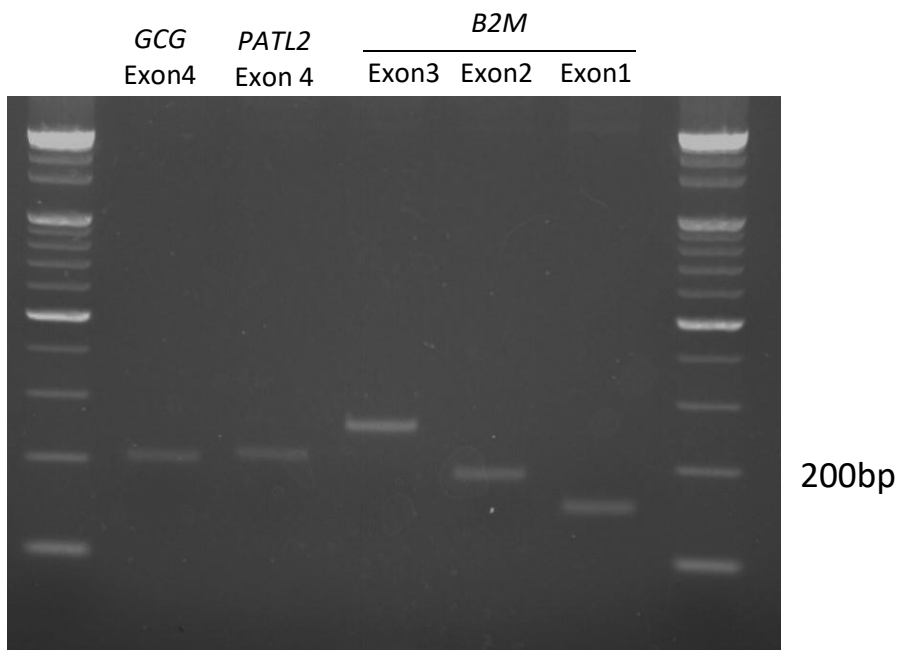

**C**

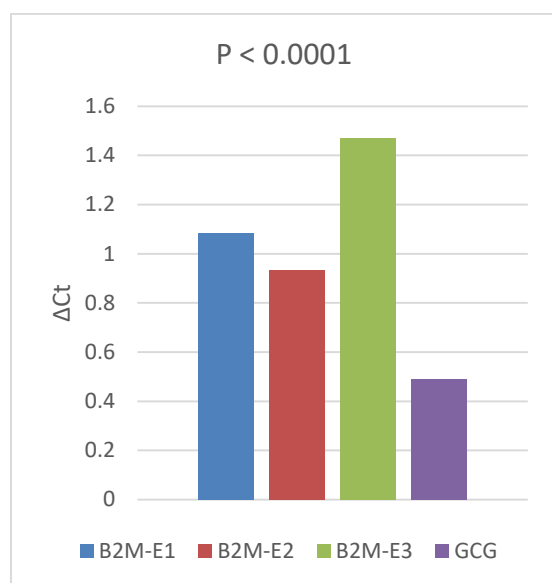

Supplement: S3 Fig — A. The results of the reverse transcription (RT)-PCR using the same primers used for the real-time PCR to estimate the levels of B2M expression in cell lines, HEK-293T (human), PK13 (pig) and NIH-3T3 (mouse) which were showed in (A) as 1, 2 and 3, respectively. n.c, negative control, L, DNA ladder. B. Result of the semi-quantitative PCR (23 cycles) to estimate the copy number of the B2M and PATL2 genes in the pig genome using the pig genomic DNA. The band intensities of the B2M exons are stronger than those of the GCG and PATL2 genes. Amplicons specific for the GCG exon 4, PATL2 exon 4, and B2M exons 1, 2, and 3 are shown. C. Results of the real-time PCR using the primers in B. E1, E2, and E3 indicate exons 1, 2, and 3. ΔCt indicates the difference in the amounts of amplicons between single copy control gene, GCG, and B2M. PATL2 was used as a house keeping gene for comparison. (PDF) [file pone.0182322.s006.pdf]

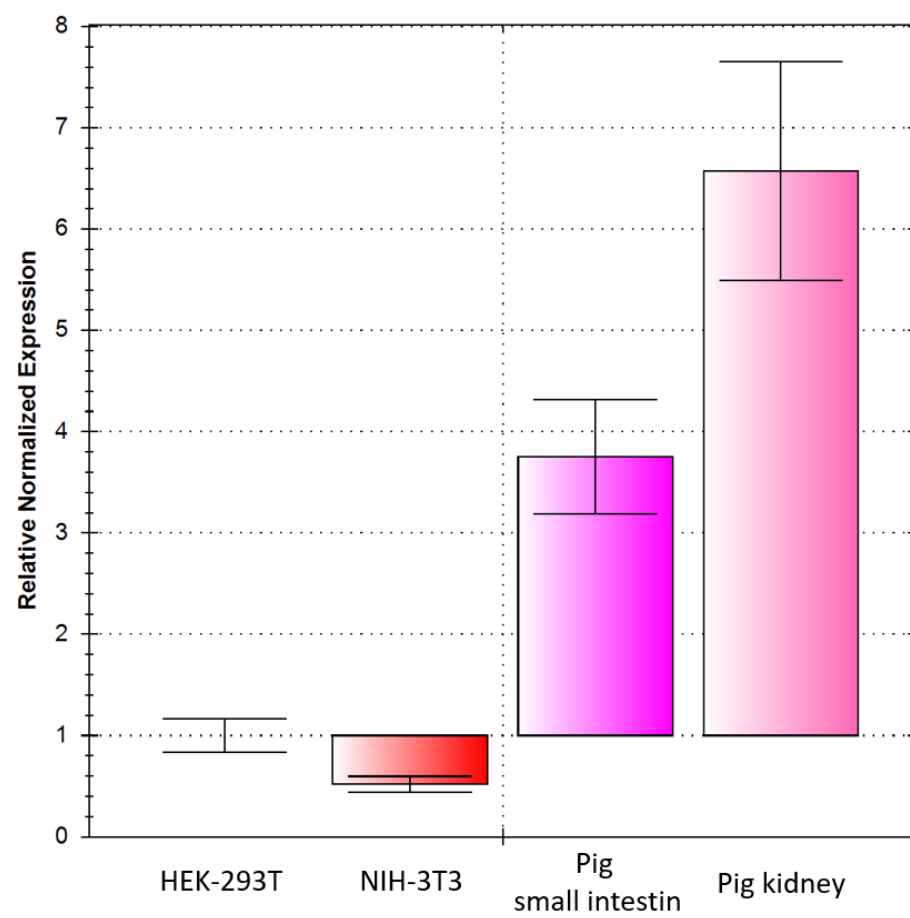

Supplement: S4 Fig — The GAPDH gene was used as a single copy gene control. Each eaction was repeated three times and the relative normalized expression level of each sample to HEK-293T cells (human) was indicated in Y-axis (ΔΔCt method). (PDF) [file pone.0182322.s007.pdf]

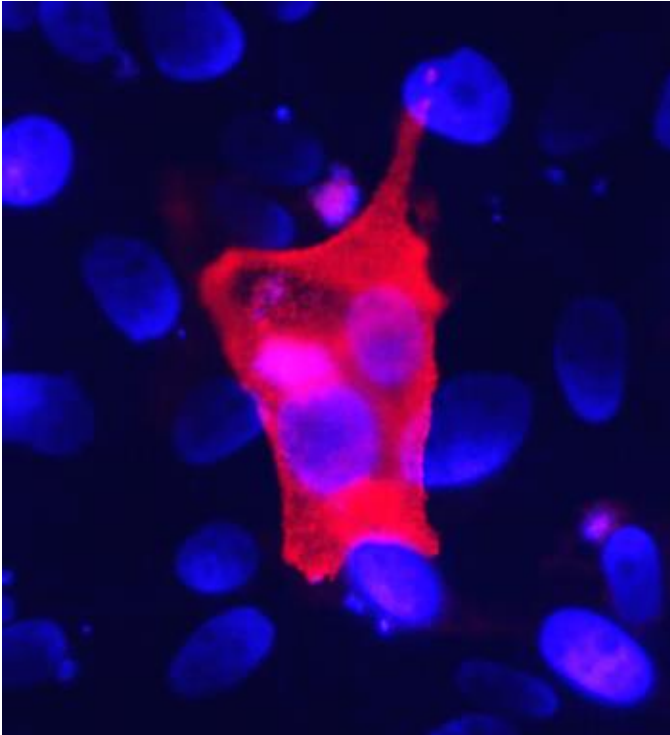

Supplement: S5 Fig — The signals, which indicate the expression of HA-B2M recombinant fusion protein (red), were observed in cytoplasm and on cell surface. The nuclei of cells (blue) were stained with 4′,6-diamidino-2-phenylindole (DAPI). (PDF) [file pone.0182322.s008.pdf]
