# Supplementary material for: β2-microglobulin gene duplication in cetartiodactyla remains intact only in pigs and possibly confers selective advantage to the species
Source: PLoS One. 2017 Aug 16;12(8):e0182322. doi: 10.1371/journal.pone.0182322 (PMC5558954; doi:10.1371/journal.pone.0182322)

Non-transfected/Alexa 568

pEGFP-N1

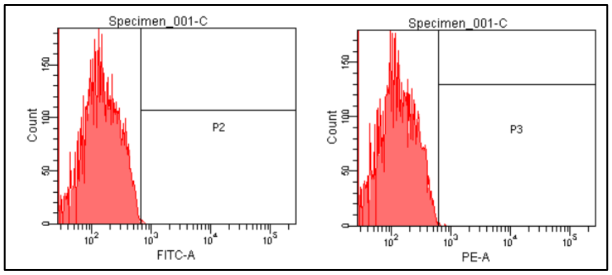

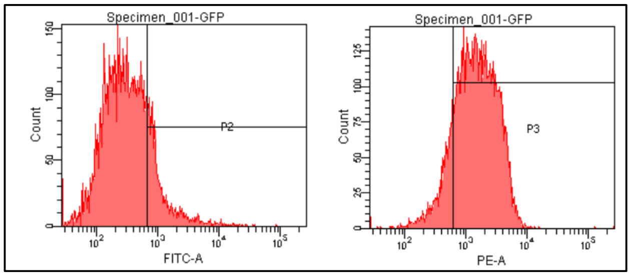

pCMV-HA/SLA class I staining/Alexa 568

Trial 1

Trial 2

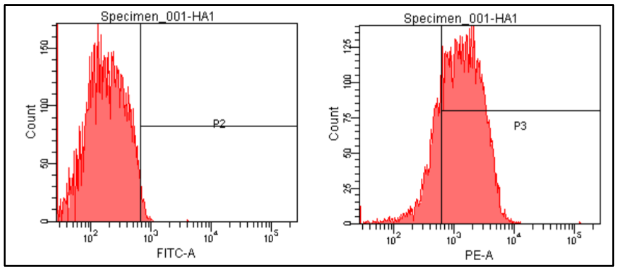

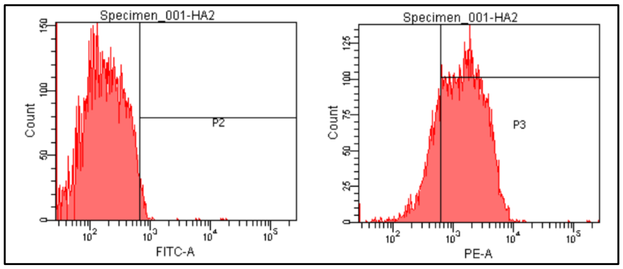

pCMV-HA-**B2M**/SLA class I staining/Alexa 568

Trial 1

Trial 2

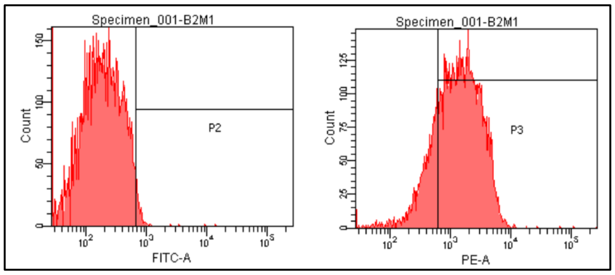

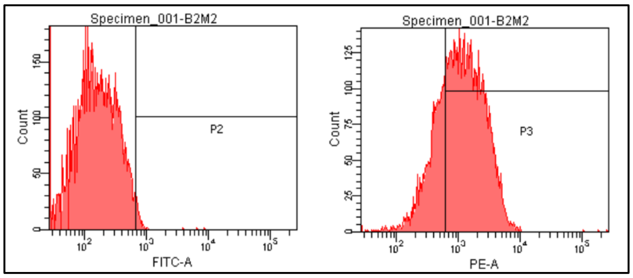

Supplement: S6 Fig — The expression construct, HA-tagged-B2M (pCMV-HA-B2M), HA tag only (pCMV-HA), and EGFP (pEGFP-N1) were transfected into cells. Pig SLA class I-specific antibodies and Alexa 568-conjugated anti-mouse IgG antibodies were used as primary and secondary antibodies, respectively. EGFP (pEGGP-N1) was used to evaluate the transfection efficiency (green GFP detected). Non-transfected cells were used as control. The analysis was performed twice for each construct. (PDF) [file pone.0182322.s009.pdf]
