# Supplementary material for: β2-microglobulin gene duplication in cetartiodactyla remains intact only in pigs and possibly confers selective advantage to the species
Source: PLoS One. 2017 Aug 16;12(8):e0182322. doi: 10.1371/journal.pone.0182322 (PMC5558954; doi:10.1371/journal.pone.0182322)
Supplement: S7 Fig — The grey box indicates the deleted amino acid in the retrotransposon sequence. Whale_retro indicates the retrotransposon copy of the B2M gene in whale. NCBI accession number: whale, XM004281255; whale retrotransposon, NW004438467; pig, L13854; cattle, BC118352; goat, XM013967395; sheep, NM001009284; horse, NM001082502; cat, NM001009876; dog, NM001284479; human, NM004048; mouse, NM009735. (PDF) [file pone.0182322.s010.pdf]

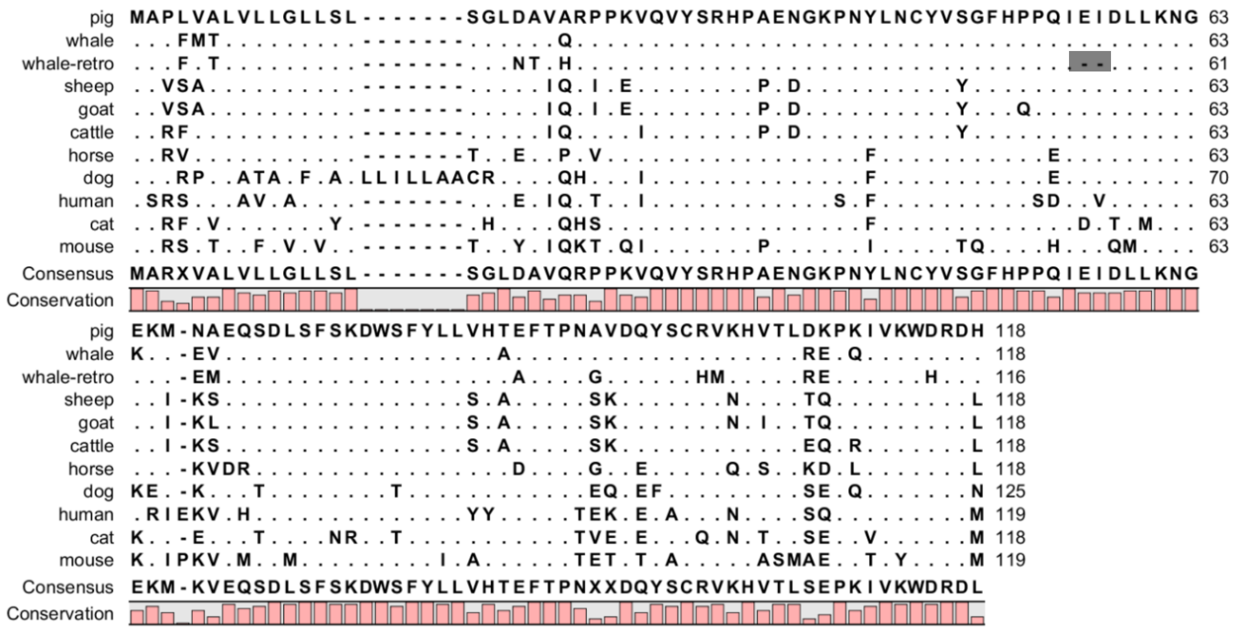
pig MAPLVALVLLGLLSL-------SGLDAVARPPKVQVYSRHPAENGKPNYLNCYVSGFHPPQIEIDLLKNG 63
whale ...FMT.........-------......Q... 63
whale-retro ...F.T.........-------...NT.H.................................--...... 61
sheep ..VSA..........-------.....IQ.I.E........P.D..........Y............... 63
goat ..VSA..........-------.....IQ.I.E........P.D..........Y...Q........... 63
cattle ..RF...........-------.....IQ....I.......P.D..........Y............... 63
horse ..RV...........-------T..E..P.V.................F...........E......... 70
dog ...RP..ATA.F.A.LLILLAACR....QH...I..............F...........E......... 70
human .SRS...AV.A....-------...E.IQ.T..I............S.F..........SD..V...... 63
cat ..RF.V.......Y.-------.H....QHS.................F.............D.T.M... 63
mouse ..RS.T..F.V.V..-------T..Y.IQKT.QI.......P......I.....TQ....H...QM.... 63
Consensus MARXVALVLLGLLSL-------SGLDAVQRPPKVQVYSRHPAENGKPNYLNCYVSGFHPPQIEIDLLKNG
Conservation
pig EKM-NAEQSDLSFSKDWSFYLLVHTEFTPNAVDQYSCRVKHVTLDKPKIVKWDRDH 118
whale K..-EV..................A...................RE.Q........ 118
whale-retro ...-EM...................A....G......HM.....RE......H... 116
sheep ..I-KS................S.A.....SK.......N....TQ.........L 118
goat ..I-KL................S.A.....SK.......N.I..TQ.........L 118
cattle ..I-KS................S.A.....SK............EQ.R.......L 118
horse ...-KVDR.................D....G..E.....Q.S..KD.L.......L 118
dog KE.-K...T........T............EQ.EF.........SE.Q.......N 125
human .RIEKV.H..............YY.....TEK.E.A...N....SQ.........M 119
cat K..-E...T....NR..T...........TVE.E...Q.N.T..SE..V......M 118
mouse K.IPKV.M..M.........I.A......TET.T.A.....ASMAE..T.Y....M 119
Consensus EKM-KVEQSDLSFSKDWSFYLLVHTEFTPNXXDQYSCRVKHVTLSEPKIVKWDRDL
Conservation
